# Detection of Invasive *Anopheles stephensi* Mosquitoes through Molecular Surveillance, Ghana

**DOI:** 10.3201/eid3003.231638

**Published:** 2024-03

**Authors:** Yaw A. Afrane, Anisa Abdulai, Abdul R. Mohammed, Yaw Akuamoah-Boateng, Christopher M. Owusu-Asenso, Isaac K. Sraku, Stephina A. Yanney, Keziah Malm, Neil F. Lobo

**Affiliations:** University of Ghana, Accra, Ghana (Y.A. Afrane, A. Abdulai, A.R. Mohammed, Y. Akuamoah-Boateng, C.M. Owusu-Asenso, I.K. Sraku, S.A. Yanney);; Ghana Health Service, Accra (K. Malm);; University of Notre Dame, Notre Dame, Indiana, USA (N.F. Lobo)

**Keywords:** *Anopheles stephensi*, malaria, invasive species, urban malaria, vector-borne infections, mosquito-borne infections, parasites, Ghana

## Abstract

The invasive *Anopheles stephensi* mosquito has rapidly expanded in range in Africa over the past decade. Consistent with World Health Organization guidelines, routine entomologic surveillance of malaria vectors in Accra, Ghana, now includes morphologic and molecular surveillance of *An. stephensi* mosquitoes. We report detection of *An. stephensi* mosquitoes in Ghana.

*Anopheles stephensi* is an invasive mosquito species originating from parts of Southeast Asia and the Arabian Peninsula (*1*). Over the past decade, *An. stephensi* mosquitoes have been expanding in range and have now been documented in several countries in Africa (*2*). First detected in Djibouti, on the Horn of Africa, in 2012, this vector has been implicated in urban malaria outbreaks (*3*). They were also detected in Ethiopia in 2016 and 2018 (*4*,*5*). *An. stephensi* mosquitoes were subsequently detected in Sudan (2016), Somalia (2019), Nigeria (2020), and Kenya (2023) (*2*,*3*,*5*–*7*). This invasive vector poses a major threat to current malaria control and elimination efforts. The ability of *An. stephensi* mosquitoes to breed in artificial containers enables them to thrive in urban areas, setting them apart from other major malaria vectors (*8*). This species can also transmit both *Plasmodium falciparum* and *P. vivax* protozoa (*1*). Although malaria is widely a rural disease, transmission in urban areas may rise because of the establishment of *An. stephensi* mosquitoes, putting ≈126 million persons at risk of malaria (*2*,*8*). The World Health Organization issued an initiative in 2022 aimed at strengthening surveillance to help stop the spread of *An. stephensi* mosquitoes in sub-Saharan Africa (*2*). Morphologic and molecular surveillance of *An. stephensi* mosquitoes were incorporated into routine entomologic surveillance of malaria vectors in the city of Accra, Ghana, after the World Health Organization initiative (*2*). This study outlines the entomologic surveillance that documents the identification of this invasive species in Ghana.

We conducted routine entomologic surveillance in 8 sites within the city of Accra, Ghana, during January 2022–July 2022 (Figure). We conducted larval sampling in all mosquito larval breeding habitats encountered in each of the sites. We recorded the total number of dips, larvae, and pupae, and we calculated the larval density as the ratio of the number of larvae collected per dip. We conducted larval sampling in the dry (February–March) and rainy (June–July) seasons of 2022. We transported larval samples to the insectary at the Department of Medical Microbiology, University of Ghana Medical School (Accra, Ghana), where we raised them into adults for morphologic and molecular species identification. We further identified members of the *An. gambiae* sensu lato, complex and sibling species by using PCR. We performed PCR amplifications to detect *An. stephensi* mosquitoes by using primers targeting the internal transcribed spacer region on the basis of on previously described protocols by Singh et al. (*9*). After PCR, were subjected 2 mosquitoes to Sanger sequencing of the internal transcribed spacer 2 regions and analyzed them on the basis of comparisons to the National Center for Biotechnology Information database.

**Figure:**
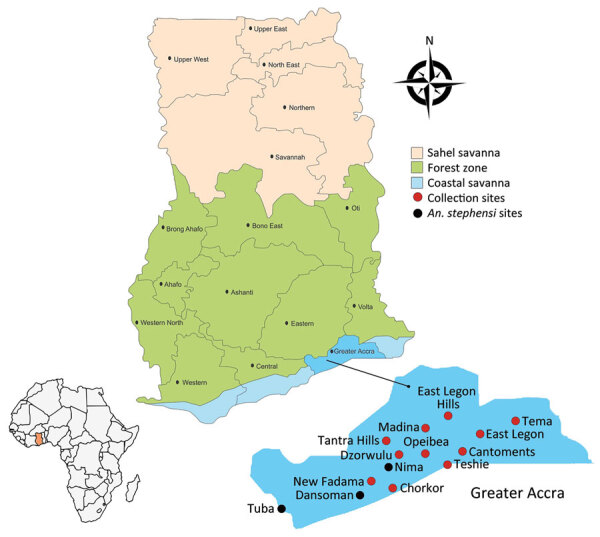
Routine entomologic surveillance sites, Accra, Ghana, January 2022–July 2022. Inset map shows location of Ghana in Africa.

We identified a total of 1,169 mosquitoes obtained from the larval sampling by using morphologic keys and PCR methods for speciation. Out of that number, 551 (47.13%) were *An. gambiae* sensu stricto, 582 (49.79%) *An. coluzzii*, and 32 (2.74%) hybrids of both species. We identified 4 samples (0.34%) as *An. stephensi* by using a modified PCR-based method by Singh et al. (*9*) and sequencing (Appendix Table 1). Results from BLAST analysis (https://blast.ncbi.nlm.nih.gov/Blast.cgi) showed that the *An. stephensi* mosquito samples had 100% sequence similarity with *An. stephensi* voucher A268 5.8S ribosomal RNA gene and internal transcribed spacer 2 (GenBank accession no. MH650999.1) (Table).

**Table:** Sequencing results of suspected *Anopheles stephensi* mosquito samples, Accra, Ghana

Sample	ITS2 contig	BLAST result†	GenBank accession no. of best match	% Identity match	Final species identification	GenBank accession no.
DN 035	283	*An. stephensi* voucher	MH650999.1	100	*An. stephensi*	OR711900
TP 002S	283	*An. stephensi* voucher	MH650999.1	100	*An. stephensi*	OR711899

*ITS2, internal transcribed spacer 2 region. †BLAST, https://blast.ncbi.nlm.nih.gov/Blast.cgi.

We found *An. stephensi* mosquitoes in larval samples from urban areas of Accra, Ghana, specifically the suburbs of Tuba, Dansoman, and Nima. We found *An. stephensi* mosquitoes breeding in dugout wells within irrigated vegetable farms and roadside ditches (Appendix Figure), habitats that are distinct from the typical ones observed in Asia and East Africa (*10*). In addition, *An. stephensi* larvae were present alongside *An. gambiae* s.s. and *An. coluzzii* mosquitoes, even though *An. stephensi* larvae are usually present alongside *Aedes* mosquitoes.

The spread of *An. stephensi* mosquitoes in Africa is thought to have occurred through land borders, air travel, or seaports. However, we discovered the mosquitoes at considerable distances from those points of entry, suggesting possible earlier introductions. Expanding surveillance efforts for *An. stephensi* mosquitoes is crucial to curbing the dissemination of this invasive species within Ghana, which could potentially elevate malaria prevalence in the city of Accra, traditionally considered a low malaria transmission zone within Ghana.

This report of the invasion of *An. stephensi* mosquitoes in Accra, Ghana, represents a major public health concern, given the heightened risk of urban malaria outbreaks. It is imperative to reinforce surveillance and response strategies in both rural and urban settings across Ghana, with specific attention directed toward *An. stephensi* mosquitoes, to mitigate the spread of this invasive species.

Appendix Additional information about detection of *Anopheles stephensi* mosquitoes through molecular surveillance, Ghana.
