# Supplementary material for: Detection of Invasive Anopheles stephensi Mosquitoes through Molecular Surveillance, Ghana
Source: Emerg Infect Dis. 2024 Mar;30(3):605–8. doi: 10.3201/eid3003.231638 (PMC10902527; doi:10.3201/eid3003.231638)
Supplement: Appendix — Additional information about detection of Anopheles stephensi mosquitoes through molecular surveillance, Ghana. [file 23-1638-Techapp-s1.pdf]

Article DOI: https://doi.org/10.3201/eid3003.231638

EID cannot ensure accessibility for supplementary materials supplied by authors. Readers who have difficulty accessing supplementary content should contact the authors for assistance.

Detection of Invasive *Anopheles stephensi* Mosquitoes through Molecular Surveillance, Ghana

Appendix

Study Sites

Sampling was conducted in 8 sites within the city of Accra, Ghana, as part of routine entomological surveillance from January 2022 to July 2022. These sites were categorized to represent different environments and socio-economic status; irrigated urban farming (IUF) sites (Tuba and Dzorwulu), lower socioeconomic (LS) sites (Nima and Chorkor), middle socioeconomic (MS) sites (Dansoman and Teshie) and high socioeconomic (HS) sites (East Legon and Cantonment). Tuba (5° 30′ 47"N 0° 23′ 16" W) and Dzorwulu (5°36′53″N 0°12′03″W) are sites where irrigated farming is practiced all year round leading to the creation of mosquito breeding sites. Socio-economic sites were classified based on their population, housing structures and the availability of proper drainage and sanitation systems. Low socioeconomic sites, Nima (5° 35′ 0″ N, 0° 12′ 0″ W) and Chorkor (5°31′39″N 0°13′55″W) are densely populated slums with poor sanitation and inadequate drainage systems. Dansoman (5° 33′ 0″ N, 0° 16′ 0″ W) and Teshie (5° 35′ 0″ N, 0° 6′ 0″ W) are middle socioeconomic sites with more standard residential structures with well-designed drainage and sanitation systems but poorly managed. High socioeconomic sites, Cantonment (5° 35′ 10″ N, 0° 10′ 35″ W) and East Legon (5°38′16.39"N, 0°9′40.33"W) have proper housing structures with good sanitation and drainage systems. Accra is the capital city of Ghana and it is the most populous. Accra lies in the coastal savannah zone of Ghana, with an annual mean temperature of 26.5°C and an average annual precipitation of 787 mm. Figure in main text (https://wwwnc.cdc.gov/EID/article/30/3/23-1638-F1.htm) shows a map of the routine surveillance sites.

Anopheles Larval Densities in Different Habitat Types across Different Sites

Ten different habitat types were encountered during the larval sampling. The highest larval density during the dry and wet seasons was observed in drainage ditches from Chorkor (9.72 larvae/dip) and swamps in Teshie (20.3 larvae/dip) respectively. Drainage ditches were consistently productive across almost all the sites in both seasons. The most productive habitat type across all the sites was drainage ditches. However, habitat types such as footprints, swamps and tire tracks also recorded low to high larval densities in some of the sites (0.25 to 20.3 larvae/dip). In Tuba, Nima and Dansoman, where *An. stephensi* mosquitoes were found, and some of the more productive habitats were drainage ditches (1.45 to 8.39 larvae/dip) and tire tracks (0.77 to 14.96 larvae/dip) (Appendix Table 2). Appendix Figure 2 shows habitats where *An. stephensi* mosquitoes were found. *An. gambiae s.l.* larval density was significantly associated with season (t = 4.14, p = 0.00).

Appendix Table 1. *Anopheles* larvae species distribution across different sites

Site	Site Category	Species, no. (%)				
		An. gambiae	*An. coluzzii*	Hybrids	*An. stephensi*	Total
Tuba	IUF	197 (61)	116 (35.9)	8 (2.5)	2 (0.6)	323 (100)
Dzorwulu		5 (31.3)	11 (68.7)	0	0	16 (100)
Nima	LS	67 (33.5)	120 (60)	12 (6)	1 (0.5)	200 (100)
Chorkor		17 (29.3)	41 (70.7)	0	0	58 (100)
Dansoman	MS	7 (7.1)	84 (85.7)	6 (6.1)	1(1.1)	98 (100)
Teshie		166 (46.62)	186 (52.2)	3 (1.2)	0	355 (100)
East Legon	HS	77 (77.7)	19 (19.3)	3 (3)	0	99 (100)
Cantonment		15 (75)	5 (25)	0	0	20 (100)
Total		551 (47.13)	582 (49.79)	32 (2.74)	4 (0.34)	1169 (100)

Appendix Table 2. *Anopheles* larval density in the dry and rainy seasons*

Habitat type	Sites/Seasons															
	Tuba		Dzorwulu		Nima		Chorkor		Dansoman		Teshie		East Legon		Cantonments	
	Dry	Wet	Dry	Wet	Dry	Wet	Dry	Wet	Dry	Wet	Dry	Wet	Dry	Wet	Dry	Wet
Man-made pond	5.15	0	0	0	0	0	0	0	0	0	0	0	0	0	0	0
Car tire	0	0	0	0	0	0	0	0	0	0	0	2.3	0	0	0	0
Drainage ditch	6.08	0	1.68	0.59	8.39	2.25	9.72	4.35	1.83	**1.45**	6.7	5.78	1.14	0.9	2.33	1.43
Footprint	0	1.6	0	3.53	0	1.97	0	6.44	0	4.52	0	5.67	0	0	0	0
Furrow	3.18	6.27	0	0	0	0	0	0	0	0	0	0	0	0	0	0
Natural pond	0	0	6.15	0	0	0	0	0	0	0	0	0	0	0	0	0
Puddle	0	4.16	0	0	0	0	0	0	0	4	0	4.06	0	0	0	0
Swamp	0	0	5.75	1.27	0	**2.67**	0	0	0	2.31	0	20.3	0	1	0	2.85
Tire track	12	14.96	0	0	0	2.69	0	3	0	3.16	0.77	8.95	0	1.52	0	1
Well	**0.96**	0	0	0	0	0	7.5	4	0	0	0	0	0	0.25	0	0

*Values in bold represent habitat types were *An. stephensi* larvae were found.

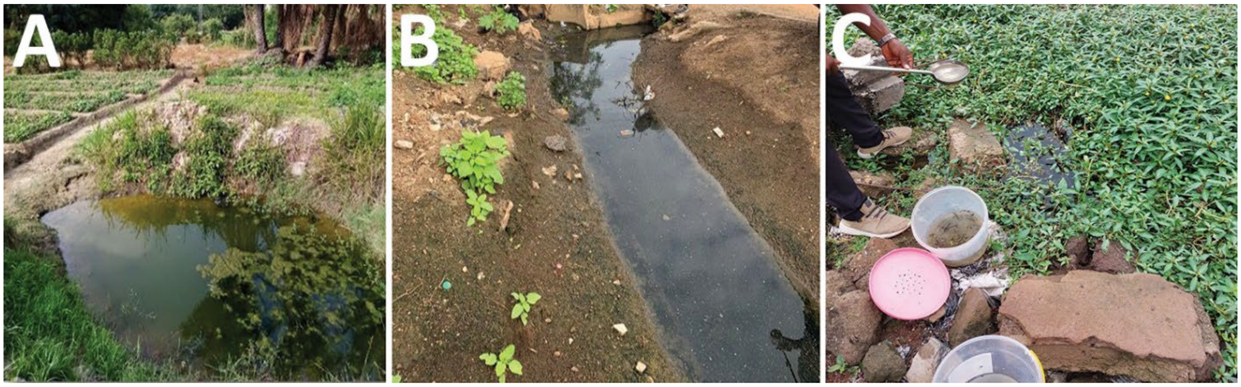

Appendix Figure. Habitats where *An. stephensi* larvae were found. A) Dug-out well (Tuba); B) drainage ditches (Dansoman); C) swamp (Nima).
